# Seed Transmission of Beet Curly Top Virus and Beet Curly Top Iran Virus in a Local Cultivar of Petunia in Iran

**DOI:** 10.3390/v9100299

**Published:** 2017-10-16

**Authors:** Ameneh Anabestani, Seyed Ali Akbar Behjatnia, Keramat Izadpanah, Saeid Tabein, Gian Paolo Accotto

**Affiliations:** 1 Plant Virology Research Center, College of Agriculture, Shiraz University, Shiraz 71441-65186, Iran; aanabestani@gmail.com (A.A.); izadpana@shirazu.ac.ir (K.I.); tabein.saeid@gmail.com (S.T.); 2 Institute for Sustainable Plant Protection, National Research Council of Italy (IPSP-CNR), 10135 Torino, Italy; Gianpaolo.Accotto@ipsp.cnr.it

**Keywords:** geminiviruses, petunia, beet curly top viruses, seed transmission, transmission rate

## Abstract

Beet curly top virus (BCTV) and beet curly top Iran virus (BCTIV) are known as the causal agents of curly top disease in beet and several other dicotyledonous plants in Iran. These viruses are transmitted by *Circulifer* species, and until now, there has been no confirmed report of their seed transmission. A percentage (38.2–78.0%) of the seedlings developed from the seeds of a petunia local cultivar under insect-free conditions showed stunting, interveinal chlorosis, leaf curling, and vein swelling symptoms, and were infected by BCTV when tested by PCR. Presence of BCTV in seed extracts of petunia local cultivar was confirmed by PCR and IC-PCR, followed by sequencing. Agroinoculation of curly top free petunia plants with a BCTV infectious clone resulted in BCTV infection of plants and their developed seeds. These results show the seed infection and transmission of BCTV in a local cultivar of petunia. Similar experiments performed with BCTIV showed that this virus is also seed transmissible in the same cultivar of petunia, although with a lower rate (8.8–18.5%). Seed transmission of curly top viruses may have significant implications in the epidemiology of these viruses.

## 1. Introduction

Curly top is a destructive disease of sugar beet and a limiting factor in the semi-arid regions of the world, such as the Middle East and the Western US. The symptoms of curly top disease (CTD) include stunted and distorted plant growth, leaf curling, vein swelling, and necrosis of hyperplasic phloem [1]. The disease affects over 300 plant species, including crops, ornamentals, and weeds from at least 44 families [1]. Since the first report from Iran in 1967 [2], curly top has been an economically important disease of sugar beet in this country. Natural infection of petunia plants with BCTV-Svr and BCTIV grown at greenhouses and street sides in Shiraz city have already been reported [3,4]. Petunia plants infected with BCTV-Svr and BCTIV showed a range of symptoms, including yellowing, interveinal chlorosis, severe leaf curling and deformation, shortening of internodes, and stunting [3,4,5].

A severe strain of beet curly top virus (BCTV-Svr (IR-SVR-86), previously known as BSCTV-(IR:86)) [6,7] and beet curly top Iran virus (BCTIV) are agents of CTD in Iran [7,8]. BCTV, belonging to the genus *Curtovirus* and BCTIV (genus *Becurtovirus*), despite having different genome sequence and organization, are similar in biological properties, such as leafhopper transmission and host range [9,10]. Whereas BCTIV is so far reported only from Iran, BCTV occurs in many regions of both the New and Old Worlds [7,11].

Natural transmission of these viruses occurs by cicadellid leafhoppers in a persistent (circulative, nonpropagative) manner. *Circulifer haematoceps*, the dominant leafhopper species found in sugar beet fields in Iran [3], is the vector of both BCTV-Svr and BCTIV in this country [10,12]. *C. tenellus*, which occurs in a low population in sugar beet fields in Iran [3], is also the vector of BCTV-Svr, whereas it is the only known vector of curly top viruses in the USA [1]. Whereas natural transmission of BCTV and related viruses occurs only by insect vector, a low rate of mechanical transmission of Beet curly top virus (BCTV) by needle injection has also been reported [1]. On the other hand, cloned genomic DNAs of geminiviruses have been widely used as infectious constructs in Agrobacterium-mediated experimental transmission (agroinoculation) of these viruses [7,9,13,14]. Until recently, there was no confirmed report of seed transmission in geminiviruses. However, maize streak virus (genus *Mastrevirus*) has been experimentally transmitted in maize through vascular puncture of the seeds [15]. More recently, Kim et al. [16], Kil et al. [17], and Kothandaraman et al. [18] reported seed transmission of sweet potato leaf curl virus (SPLCV), tomato yellow leaf curl virus (TYLCV), and mung bean yellow mosaic virus (MBYMV), three distinct begomoviruses in sweet potato (*Ipomoea batatas*), tomato (*Solanum lycopersicum*), and black gram (*Vigna mungo*), respectively. In the present paper, we provide evidence for natural and experimental seed transmission of BCTV-Svr, a curtovirus, and BCTIV, a becurtovirus, in a local cultivar of petunia. A preliminary report on seed transmission of these viruses has been presented previously [5].

## 2. Materials and Methods

### 2.1. Sources of Petunia Seeds and Virus Isolates

Seeds of petunia (*Petunia X hybrida*), known as the Moaatar (fragrant) variety, used in this study, were from a batch provided by a local florist in Shiraz, Iran (referred to as local cultivar in this study). Purple Classic Spreading Petunia (PCSP) cultivar (*Petunia X hybrida*) from Pan American Seed Company, USA, was also used in certain experiments. Seeds of PCSP plants which tested negative for curly top viruses (BCTV-Svr or BCTIV) by PCR were used as negative control. Plants were grown in a glass-sided insect-free greenhouse at 20–28/16–20 °C (day/night), and sprayed every fortnight with Confidor (Bayer, Leverkusen, Germany) and Pyriproxyfen (Hef. Chem. Co., Tehran, Iran) insecticides. In addition, yellow sticky cards were also used, both to capture and monitor the presence of any insects [19], and to be sure that no insects were able to enter the greenhouse.

Constructs of a BCTV-Svr infectious clone (pBin-1.7BCTV-Svr) [14] and a BCTIV infectious clone (pBin-1.4BCTIV) [9], in the binary transformation vectors pBin20 and pBin19, respectively, were used to agroinoculate curly top-free (healthy) petunia plants.

### 2.2. Seed Transmission Experiments

Seeds of the local cultivar were sown in an insect-free greenhouse in Plant Virology Research Center (PVRC) in Shiraz University, Shiraz, and the seedlings were checked daily for symptoms. They were tested for infection by BCTV and BCTIV by PCR, using primer pairs specific for each virus (Table 1). In a different experiment, to evaluate vertical transmission of BCTVs through seed, eight symptomatic and two symptomless petunia plants developed from the seeds of the local cultivar were selected, and allowed to grow and produce seeds inside an insect-free greenhouse. Seeds were collected separately and planted again for three generations. In each cycle, seedlings were examined for infection with curly top viruses by PCR using viral specific primer pairs (Table 1).

To explore the possibility of virus transmission through the seed obtained from plants infected following agroinoculation, curly top free petunia seedlings were separately inoculated with BCTV-Svr and BCTIV infectious clones. *Agrobacterium tumefaciens* cultures carrying the infectious clone of BCTV-Svr or BCTIV were grown at 28 °C for 36–48 h. Five seedlings of local cultivar and five seedlings of PCSP cultivar were inoculated at 8–10 leaf stage each, by injection of 100 µL of culture (OD_600_ = 1.0) at several leaf nodes. Five non-inoculated plants of each cultivar were also kept as controls in the same greenhouse. All plants were maintained in the greenhouse under insect-free conditions for seed production. Young leaves were sampled 21 days post-inoculation (dpi) and the presence of BCTV-Svr or BCTIV was checked by PCR, as described below. When plants produced seed, DNA was extracted from seeds and analyzed.

### 2.3. DNA Extraction, PCR Analysis and Sequencing

Total DNA was extracted from 200 mg of plant leaf tissues and 100 mg of petunia seeds (approximately 1200 seeds) using a modified cetyltrimethyl ammonium bromide (CTAB) procedure originally described by Gawel and Jarret [20]. Leaves were homogenized using a mortar and pestle in 700 µL of extraction buffer (3% CTAB, 100 mM Tris-HCl, pH 8, 10 mM EDTA, pH 8.0, 1.4 M NaCl) preheated at 65 °C. The homogenate was transferred into a centrifuge tube, and incubated for 30 min at 65 °C. After addition of equal volume of chloroform and centrifugation, the upper phase was transferred to a new tube, and nucleic acids precipitated by adding equal volume of isopropanol and centrifugation. The pellet was washed with 70% ethanol, and dissolved in 60 µL of sterile double-distilled water.

PCR assays were carried out in 25 µL reaction mixtures containing 2 µL DNA template (approximately 50–60 ng DNA of leaf tissues or DNA from approximately 40 seeds), 1.5 mM MgCl_2_, 0.2 mM of each dNTP, 1 µM of each primer, and 1.5 U of *Taq* DNA polymerase (Cinagen, Tehran, Iran) in the reaction buffer provided by the same source. The mixture was heated for 3 min at 95 °C, and subjected to a 30 cycle-PCR program of 30 s at 94 °C, 45 s at 54 °C for BCTV-Svr (49 °C for BCTIV), and 1 min at 72 °C. The final cycle was followed by 10 min at 72 °C. Total DNA extracted from healthy plants or seeds were used as negative control in all PCR tests. The reaction mixture was then loaded directly onto a 1% agarose gel, stained with ethidium bromide, and visualized by UV light. The primer pairs BCTV-Svr358^V^/Svr877^C^ and BCTIV474^V^/1155^C^ were used to amplify the BCTV-Svr 520-bp and BCTIV 682-bp DNA fragments, respectively (Table 1).

The amplicons produced from one BCTV-Svr-agroinoculated plant, seeds of such a plant, and one naturally BCTV-Svr-infected plant, were purified using PCR purification kit (Qiagen, Hilden, Germany) according to manufacturer’s recommendation, and sequenced (Bioneer, Daejeon, South Korea). The obtained sequences were compared to the available sequences in the GenBank database (www.ncbi.nlm.nih.gov/genbank/) to confirm the identity of amplified fragments using BLAST tool. DNA sequences were aligned and compared to other sequences in the GenBank by ClustalX version 1.83, University College Dublin, Dublin, Ireland. Similarity percentages were calculated using Mega 6 programs [21].

### 2.4. Immunocapture PCR

Immunocapture PCR (IC-PCR) was used to examine the possible presence of BCTV-Svr in petunia seeds and seedlings. A polyclonal antibody against an Iranian isolate of BCTV [22] was used to capture BCTV-Svr particles. The 0.2 mL tubes were coated with 100 µL of 0.5 µg/mL antibodies in blocking buffer (137 mM NaCl, 2.7 mM KCl, 10 mM Na_2_HPO_4_, 1.8 mM KH_2_PO_4_, 2% polyvinylpyrrolidone, and 3% skimmed milk) and incubated overnight at 4 °C. The tubes were then washed three times with 200 µL of PBS washing buffer (137 mM NaCl, 2.7 mM KCl, 10 mM Na_2_HPO_4_, 1.8 mM KH_2_PO_4_, 0.05% Tween-20). Leaves and seeds were ground in five volumes of sample extraction buffer (PBS washing buffer plus 2% PVP) and centrifuged at 5000 *g* for 3 min at 4 °C. Aliquots of 100 µL of plant extracts were transferred to the tubes and incubated at 4 °C overnight. The tubes were then washed with PBS washing buffer. A mixture of PCR reaction containing the BCTV-Svr358^V^/877^C^ primer pair (Table 1) was added to each tube. The mixture was heated for 5 min at 95 °C to release the viral DNAs from the particles, and then subjected to a 30 cycle-PCR program as described above. The PCR products were then loaded directly onto a 1% agarose gel. After electrophoresis, the gels were stained with ethidium bromide and visualized with UV light.

### 2.5. DIG Southern Blot Hybridization Analysis

A non-radioactive (DIG-labeled) BCTV-Svr DNA probe was prepared using a PCR ELISA, DIG labeling, kit (Roche, Basel, Switzerland) according to manufacturer’s recommendation. The primer pair BCTV-Svr358^V^/877^C^ was used for production of a digoxigenin-labelled BCTV-specific probe. Total DNA (approximately 4 µg) was electrophoresed in 1% agarose gel, and blotted onto a positively charged membrane (Roche, Switzerland). Total DNA from healthy PCSP plants and seeds were used as negative controls. The DNA was cross-linked to the membrane using a UV source (HeroLab, Ludwig-Wagner, Germany). Prehybridization was carried out in 10 mL of 0.25 M phosphate buffer, pH 7.2, containing 7% SDS and 1 mM EDTA at 42 °C for 1 h. The membrane was hybridized at 42 °C overnight, after adding 25 ng/mL probe along with phosphate buffer. Membrane was then washed twice with washing buffer (150 mM NaCl, 100 mM maleic acid, and 0.33% Tween-20), once at room temperature, and once at 68 °C. DIG label was serologically detected using anti DIG-alkaline phosphatase conjugated and NBT/BCIP substrate, according to the manufacturer’s instructions, for 1 h at room temperature [23].

### 2.6. Seed Transmission Rate of BCTV-Svr and BCTIV

To compare seed transmission rate of BCTV-Svr and BCTIV in petunia, seeds of local cultivar (see above) were planted in a pot. Seeds of PCSP cultivar, proved to be free from BCTV-Svr and BCTIV by PCR, were also planted as negative control in another pot. Emerging seedlings at the 4-leaf stage were transferred to new pots (one seedling per pot), and maintained in the greenhouse under insect-free conditions, until flowering and seed production. Three separate experiments were performed with 34, 50, and 70 seedlings of the local cultivar, and 23, 56, and 70 seedlings of PCSP cultivar, respectively. The seedlings were evaluated for symptom appearance. Leaf tissue sample of each seedling was collected and tested for BCTV-Svr and BCTIV infection by PCR. In the first experiment, leaf tissue samples were collected at 4–6 leaf stage, whereas in the second and third experiments, sampling was done just before flowering.

## 3. Results

### 3.1. Detection of BCTV-Svr and BCTIV in Petunia Seeds and Their Developed Symptomatic Plants

**Figure 1:**
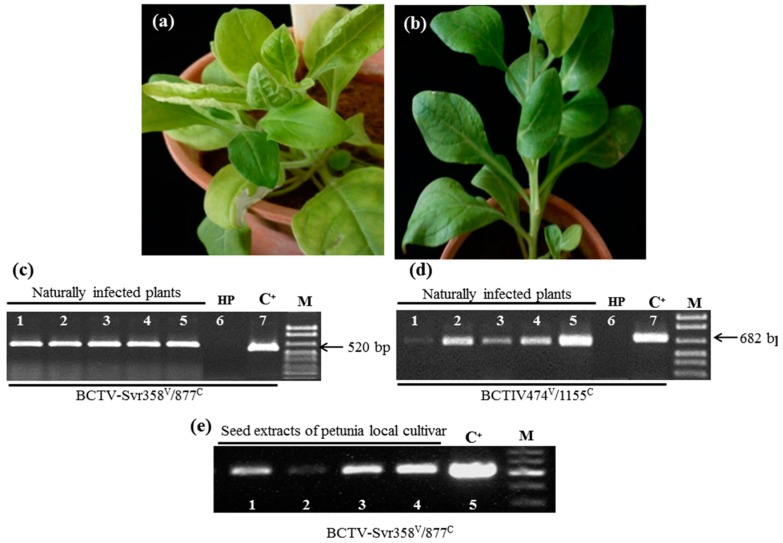
Detection of beet curly top virus (BCTV-Svr) and beet curly top Iran virus (BCTIV) in infected petunia plants. (**a**) Interveinal chlorosis, vein swelling and severe leaf curling in a BCTV-Svr infected petunia plant developed from the seed of the local cultivar; (**b**) non-symptomatic curly top free plant of local cultivar; (**c**,**d**) agarose gel electrophoresis analysis of PCR products of BCTV-Svr and BCTIV amplified from total DNA extracts of naturally infected petunia plants by specific primer pairs as indicated below each panel; (**e**) agarose gel electrophoresis analysis of PCR products of BCTV-Svr amplified from total DNA of seed extracts of petunia local cultivar by specific primer pairs as indicated below the panel. C^+^ = Positive control: cloned DNA of BCTIV (pBin-1.4-BCTIV in D) and BCTV-Svr (pBin-1.7 BCTV-Svr in E); HP = healthy (curly top free) plant. M = Marker (DNA ladder mix, Fermentas, Waltham, MA, USA).

Germination rate of petunia local cultivar seeds sown in an insect-free greenhouse in Shiraz University was approximately 50%. However, only 20 out of 50 seedlings remained strong enough at 4–8 leaf stage to allow DNA extraction from their leaves. A number of plants (see seed transmission rate section) developed from these seeds, when grown to the flowering stage, showed a range of symptoms, including interveinal chlorosis, severe leaf curling, vein swelling, and stunting (Figure 1a), whereas some others remained asymptomatic (Figure 1b). When extracts obtained from the leaves of symptomatic plants were analyzed by PCR for the presence of BCTV-Svr and BCTIV, most plants showed the presence of BCTV-Svr (producing a 520 bp fragment, Figure 1c) and some plants also showed BCTIV (682 bp product, Figure 1D). No viral DNA was amplified from asymptomatic plants (Figure 1c,d, lane 6). Presence of BCTV-Svr in seed extracts of petunia local cultivar was confirmed by PCR. The 520 bp fragment of BCTV-Svr genome was amplified from seed extracts using BCTV-specific primers (Table 1), but no BCTIV fragment was amplified from the same seeds when BCTIV-specific primers were used (Figure 1e).

### 3.2. Vertical Transmission of BCTV-Svr and BCTIV through Three Generations of Petunia

**Figure 2:**
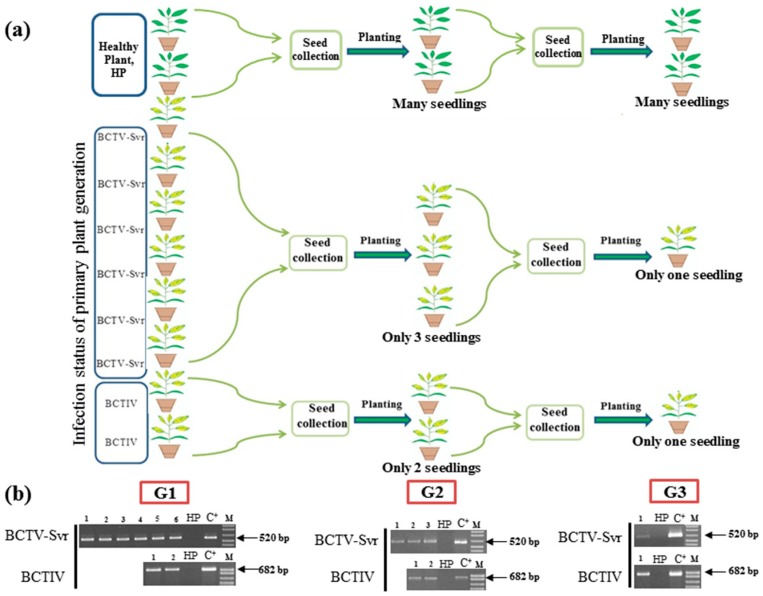
Vertical transmission of BCTV-Svr and BCTIV through three generations of naturally infected petunia plants of local cultivar. (**a**) Schematic representation of an experiment on vertical transmission of BCTV-Svr and BCTIV through three generations (G1, G2 and G3). Healthy plants are shown with green leaves and those from symptomatic infected plants are shown in yellow color; (**b**) agarose gel electrophoresis analysis of PCR products of BCTV-Svr and BCTIV amplified from total DNA extracts from each plant in each generation using specific BCTV-Svr358^V^/877^C^ or BCTIV-474^v^/1155^c^ primer pair. C^+^ = Positive control: cloned DNA of BCTV-Svr (pBin-1.7 BCTV-Svr) or of BCTIV (pBin-1.4-BCTIV). HP = healthy (curly top free) plant. C^−^ = Negative control: distilled water. M = Marker (DNA ladder mix, Fermentas).

Transmission of BCTV-Svr and BCTIV by the seed was tested through three generations in the local cultivar of petunia under insect-free conditions. Ten plants (generation 1, G1) developed from seeds of petunia local cultivar, including eight symptomatic and two symptomless plants, were selected for examination of BCTV-Svr and BCTIV vertical transmission, and checked by PCR for the presence of these viruses. Out of eight plants showing curly top symptoms, six and two plants were infected by BCTV-Svr and BCTIV, respectively. The remaining two symptomless plants were confirmed to be free of curly top viruses by PCR, and used as healthy control (Figure 2a). All plants were maintained in an insect-free greenhouse until seed production. Many flowers of the virus-infected plants failed to produce capsule or seed, whereas abundant seed was produced by healthy (curly top free) plants. In the second generation, only five symptomatic seedlings (generation 2, G2) were obtained from the few seeds collected from eight infected G1 plants, of which three and two were infected with BCTV-Svr and BCTIV, respectively. In the third generation (G3), only two symptomatic progeny plants, each infected with one of the viruses, were obtained (Figure 2a). The germination rate of seeds derived from infected plants was reduced, indicating an influence of virus infection on seed germination. In each generation, presence of curly top viruses in seedlings developed from seeds was verified by PCR (Figure 2b). No PCR amplicons were obtained with petunia seeds of the healthy plant used as control. Comparison of nucleotide sequence of PCR products (520 bp fragments) amplified from infected seed extracts and seedlings with the corresponding region of the curtoviruses available in GenBank using MEGA 6 program [21], showed that this fragment had the highest sequence identity (98–99%) with the corresponding region of BCTV-Svr [IR-SVR-86] (X97203). As BCTIV infection rate was very low, sequencing of BCTIV PCR products amplified from extracts of seedlings was not performed.

### 3.3. Presence of Virion Particles in the Seedlings and Seeds

**Figure 3:**
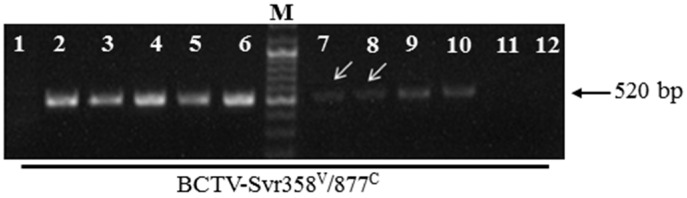
Agarose gel electrophoresis analysis of immunocapture PCR products using a BCTV antibody and specific BCTV-Svr358^V^/877^C^ primer pair. Lane 1, extract of leaf tissue of healthy plants of local cultivar as negative control; lanes 2–6, extracts of leaf tissue of naturally infected plants of local cultivar; lanes 7 and 8, extracts of seeds of naturally infected plants of local cultivar; lanes 9 and 10 extracts of seeds of BCTV-Svr-agroinoculated plants of PCSP cultivar, lanes 11 and 12 extract of seeds developed from healthy plants of PCSP cultivar. M = Marker (DNA ladder mix, Fermentas).

**Figure 4:**
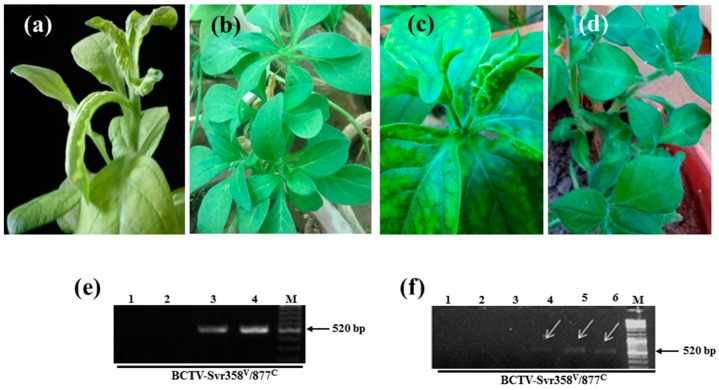
Detection of BCTV-Svr in agroinfected petunia plants and their developed seeds. (**a**) Severe curly top symptoms in a BCTV-Svr-agroinoculated plant of local cultivar; (**b**) curly top free (healthy) plants of local cultivar; (**c**) a BCTV-Svr-agroinoculated plant of PCSP cultivar showing interveinal chlorosis, leaf curling and vein swelling; (**d**) healthy plants of PCSP cultivar; (**e**,**f**) agarose gel electrophoresis analysis of IC-PCR products using a BCTV antibody and the BCTV-Svr358^V^/877^C^ primer pair (Table 1); (**e**) lanes 1 and 2, extracts of leaf tissue of healthy plants of PCSP cultivars as negative controls; lanes 3 and 4, extracts of leaf tissue of BCTV-Svr-agroinoculated plants of local and PCSP cultivars, respectively; (**f**) lanes 1–3, extracts of seeds of healthy plants of local cultivar as negative controls; lanes 4 and 5 extracts of seeds of BCTV-Svr-agroinoculated plants of local cultivar; lane 6, extract of seeds of BCTV-Svr-agroinoculated plants of PCSP cultivar. M = Marker (DNA ladder mix, Fermentas).

Presence of virus particles in leaf tissue of naturally infected plants and their seeds was examined by IC-PCR. BCTV-Svr particles were captured in extracts from leaves and seeds using a polyclonal antibody against BCTV. The captured particles were then used for PCR analysis. DNA amplification using the specific primer pair for BCTV resulted in PCR products of the expected size in naturally infected plants of local cultivar (Figure 3, lanes 2–6), and their developed seeds (Figure 3, lanes 7 and 8), suggesting the presence of whole virus particles in the seedlings and seeds. IC-PCR of seed extract gave faint bands on gels (Figure 3, lanes 7 and 8), suggesting low virus concentration. No virus was detected in control healthy plants (Figure 3, lane 1) and their seeds (Figure 4f, lanes 1–3). BCTIV was not detected in the petunia seeds in any IC-PCR experiment. Nevertheless, the virus was present and caused symptoms in some seedlings developed from seeds of infected petunia. This may be due to the lower percentage of seed infection and/or lower concentration of BCTIV in the infected seeds.

### 3.4. BCTV-Svr Infection of Seeds in Agroinfected Petunia Plants

To investigate the seed transmission of BCTV-Svr and BCTIV in agroinfected petunia plants, five petunia seedlings of local cultivar and five seedlings of PCSP cultivar were agroinoculated separately with an infectious clone of either BCTV-Svr or BCTIV. Typical symptoms of curly top disease, such as leaf curling, vein swelling, and stunting, developed in all plants of both cultivars (totally 20 plants) 2–3 weeks post inoculation (Figure 4a,c). The non-inoculated control plants of either cultivar were not symptomatic (Figure 4b,d). BCTV-Svr was detected in agroinoculated plants of both cultivars by PCR and IC-PCR (Figure 4e, lanes 3 and 4). Most BCTV-Svr-agroinoculated plants died before flowering, however, in those that produced flowers, only a few seeds were produced. Such seeds in both cultivars showed virus infection in IC-PCR (Figure 3 lanes 9–10 and Figure 4f, lanes 4–6). No IC-PCR products were obtained with petunia seeds of curly top free (healthy) plants used as controls (Figure 3 lanes 11–12 and Figure 4f, lanes 1–3). These results confirmed that agroinoculation of curly top free (healthy) plants with BCTV-Svr can result in seed infection in both petunia cultivars. Plants infected by BCTIV (as determined with PCR) did not die, and no virus DNA was detected in their seeds.

### 3.5. Southern Blot Analysis of BCTV-Svr-Infected Plants

**Figure 5:**
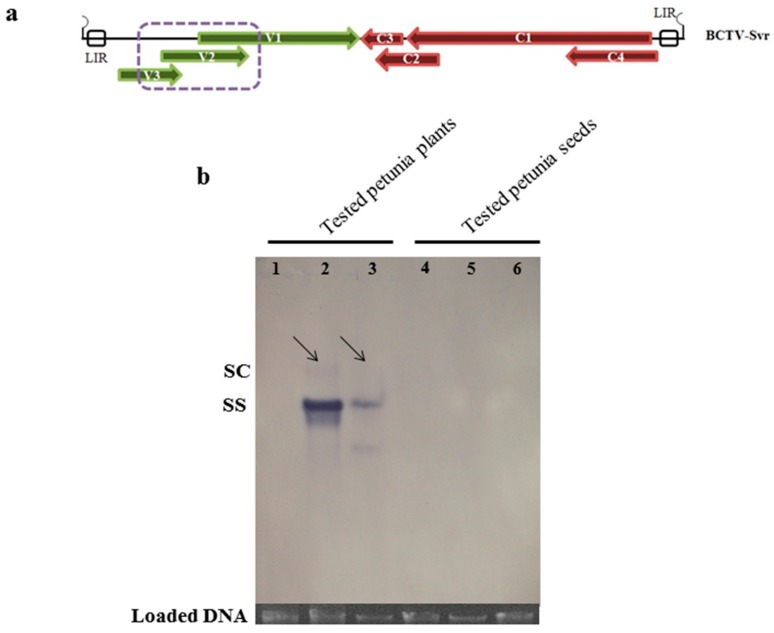
(**a**) Linearized diagram of BCTV-Svr genome. Arrows indicate coding sequences and purple dotted box indicates the region used for preparing probe for PCR ELISA (DIG labeling) and BCTV-Svr358^V^/877^C^ primer pair; (**b**) Southern blot hybridization of total DNA extracted from: a healthy PCSP plant as negative control (lane 1), an agroinoculated PCSP plant as positive control (lane 2), an infected plant developed from seed of local cultivar (lane 3), seeds of healthy PCSP used as negative control (lane 4), seeds of agroinoculated PCSP cultivar (lane 5) and seeds of naturally infected local petunia cultivar (lane 6). Loaded DNA of each sample stained with ethidium bromide is shown at the bottom of the blot panel. sc (BCTV-Svr supercoil double-strand DNA, which is shown by the arrows); ss (BCTV-Svr single-strand DNA).

Southern blot analysis of BCTV-Svr DNAs extracted from naturally infected petunia plants of local cultivar at 6 leaf stages, agroinfected petunia plants of PCSP cultivar at flowering stage (as positive control), and their developed seeds, was carried out using a cognate specific DIG-labeled DNA probe (Figure 5a). Total DNA extracted from healthy plants and seeds of PCSP cultivar were used as negative controls. Both ssDNA and dsDNA replicative form [24] of the virus were visible when DNA extracted from naturally and agroinfected plants of both cultivars were assayed (Figure 5b, lanes 2 and 3), whereas viral DNA was not visualized in extracts from the seeds (Figure 5b, lanes 5 and 6).

### 3.6. Seed Transmission Rate of BCTV-Svr and BCTIV

To estimate seed transmission rates of BCTV-Svr and BCTIV in petunia, seedlings of local cultivar were analyzed by PCR in three separate experiments. In the first experiment, 34 plants were tested at 4–6 leaf stage, resulting in 13 (38.2%) and three plants (8.8%) positive for BCTV-Svr and BCTIV, respectively. One plant (2.9%) was infected by both viruses (Table 2). At this stage, PCR positive petunia plants did not show any symptoms. None of the 23 PCSP seedlings used as controls showed infection by either BCTV-Svr or BCTIV. In the second and third experiments, petunia plants developed from seeds of the local cultivar were examined by the same method, but at flowering stage. In these experiments, infection efficiencies of these viruses were 70.0 and 78.0% for BCTV-Svr, and 10.0 and 18.5% for BCTIV, respectively (Table 2). Only five out of 120 plants (approximately 4%), tested in the second and third experiments had mixed infection by both viruses. At this flowering stage, most of PCR positive petunia plants showed interveinal chlorosis, and a few of them showed also vein swelling and leaf curling, as shown in Figure 1B. All the 126 PCSP petunia seedlings remained symptomless and free from BCTV-Svr and BCTIV, as verified by PCR (Table 2).

## 4. Discussion

As shown in the experiments summarized in Table 2, when using petunia symptomatic plants of the local cultivar grown in an insect-free greenhouse, both BCTV-Svr and BCTIV were transmitted via seed, whereas control plants of PCSP cultivar grown under the same conditions remained virus-free, despite susceptibility to the viruses.

In sugar beet, Bennet [1] could not demonstrate BCTV seed transmission, despite the presence of the virus in sugar beet seeds. There are, however, some unconfirmed reports on seed transmissibility of certain geminiviruses, including BCTV [25] and abutilon mosaic virus [26]. More recently, however, it was shown that sweet potato leaf curl virus, tomato yellow leaf curl virus (TYLCV), and MBYMV, three distinct begomoviruses, can be transmitted via seeds in sweet potato, tomato, and black gram cultivars, respectively [16,17]. There are conflicting reports about the seed transmission of TYLCV to other hosts. While it has been reported that TYLCV is seed-transmissible in soybean (*Glycine max*) [27], no evidence of seed transmissibility of TYLCV in the experimental host *Nicotiana benthamiana* was found [28]. In the present study, BCTV-Svr was detected in seeds of naturally infected petunia plants (local cultivar) and their progeny plants, by molecular and serological tests. Because very small size of petunia seeds was a limiting factor for using single seed-tests, such as tissue blot hybridization, presence of viral particle in petunia seeds was tested by IC-PCR. However, lower efficiency of IC-PCR (Figure 3 and Figure 4f) compared to regular PCR (Figure 1e) may suggest that only a small portion of the virus in the seed is in the form of whole particles. In Southern blots, there was no signal when 4 µg of DNA extracted from seeds was used, whereas both ssDNA and dsDNA forms were detected by this assay in seedlings of naturally infected petunia plants developed from such seeds (Figure 5, lane 3). The results of Southern blot analysis revealed a high concentration of the virus DNA in the leaf tissues of young seedlings originated from infected seeds of local cultivar, and also in agroinfected petunia plants of the PCSP cultivar. Failure to detect viral DNA in Southern blots from seeds that were PCR-positive indicates a low concentration of virus in these organs. Southern blot assays, and a look at the results as a whole, indicate very low concentrations of viral genome in the seeds. Kim et al. [16] showed that the SPLCV in young sweet potato seedlings developed from infected seeds was too low to be detectable by Southern blot assay, but in the later stages, replicating forms of SPLCV DNA were detected by the same method. This suggests some form of latency in virus cycle when transmitted via seed. Similar conclusions can be drawn from our results. However, given that the seeds of mung beans are large enough, confirmation of the presence of the MBYMV virion particles in a single mung bean seed was possible by performing DAS-ELISA and ISEM experiments [18].

Seed infection apparently had a detrimental effect on the seed and plant development. In this study, it was observed that many flowers of infected plants failed to produce capsule and/or seeds, and some even died before flowering. Natural incidence of BCTV-Svr in seedlings developed from the seeds of the petunia local cultivar was higher (38.2–78%) than BCTIV (8.8–18.5%), suggesting a higher transmission efficiency for one virus compared to the other. However, in a vertical transmission experiment in three generations, very few seeds were obtained, indicating a strong detrimental effect of virus infection. Considering that several factors may affect transmission of plant viruses through seeds [29,30], it is possible that the growing conditions used to produce the original batch of seed were quite different from those in the insect-free glasshouse employed in our experiments. Also, the development of seed may be affected by the time of plant infection.

Seed transmission may provide a very effective means of introducing virus into a crop at an early stage, resulting in randomized foci of primary infection, from which it may spread throughout the planting by other means [31,32]. The data presented in this study provide evidence for seed transmission of curly top viruses in petunia.

Seed transmission of viruses may be, in part, responsible for their transmission over long distances, including intercontinental spread. It becomes even more important when considering the wide host range of BCTV and BCTIV, which includes several plant families. Since the first detailed molecular description of BCTV in Iran [7], it was clear that this virus must have moved intercontinentally, either from America to the Middle East, or vice versa. Further studies will clarify if BCTV can be seed transmitted in beet, but even if the seed transmission is through a different species, such as petunia, once in another continent, the virus can move to beet in the presence of its vector. The fact that, in the case of BCTIV, our results suggest a low seed transmission rate, may in part explain why its geographical distribution is, so far, limited to the Middle East.

Further studies are needed to explore seed transmissibility of these viruses in other hosts. The outcome of this study, and recent reports on seed transmission of some other geminiviruses [16,17], could answer questions of how these viruses get around the world by plant materials, in addition to accidental movement of their viruliferous vectors.

## Figures and Tables

**Table 1:** Details of specific primers used in this study.

Primer	* Nucleotide Position	Sequence from 5′ to 3′	Expected Product Size (bp)
BCTV-Svr358^V^	358–383	GTGGATCAATTTCCAGACAATTATC	520
BCTV-Svr877^C^	853–877	CCCCATAAGAGCCATATCAAACTTC	
BCTIV474^V^	474–493	TACAAGAAGTATGGCGGTTC	682
BCTIV1155^C^	1134–1155	AAGAATAGCATTCTCCTTCAC	

* Nucleotide position of Iranian isolate of BCTV-Svr and BCTIV from the GenBank database (accession numbers X97203 and JQ707939, respectively). ^C^ Complementary-sense strand primer. ^V^ Virion-sense strand primer.

**Table 2:** PCR analysis of petunia seedlings developed from seeds under insect-free conditions of two varieties (a local cultivar and Purple Classic Spreading Petunia, PCSP) for BCTV-Svr and BCTIV infection in three different experiments in two sampling (either 4–6 leaf or flowering) stages.

Experiment	Sampling Stage	Petunia Cultivar	Infected/Tested (Percentage of Infection)		
			BCTV-Svr	BCTIV	Mixed Infection
1	4–6 leaf	local	13/34 (38.2%)	3/34 (8.8%)	1/34 (2.9%)
		PCSP	0/23 (0%)	0/23 (0%)	0/23 (0%)
2	flowering	local	39/50 (78.0%)	5/50 (10.0%)	2/50 (4.0%)
		PCSP	0/56 (0%)	0/56 (0%)	0/56 (0%)
3	flowering	local	49/70 (70.0%)	13/70 (18.5%)	3/70 (4.2%)
		PCSP	0/70 (0%)	0/70 (0%)	0/70 (0%)
